# Noninvasive carbon dioxide monitoring in pediatric patients undergoing laparoscopic surgery: transcutaneous *vs*. end-tidal techniques

**DOI:** 10.1186/s12887-023-03836-2

**Published:** 2023-01-14

**Authors:** Weitao Wang, Zhifa Zhao, Xinjie Tian, Xinggang Ma, Liang Xu, Guanglin Shang

**Affiliations:** 1 Department of Anesthesiology, Shenzhen Children’s Hospital, China Medical University, Shenzhen, China; 2 Department of Stomatology, Shenzhen Children’s Hospital, China Medical University, Shenzhen, China; 3 Department of Anesthesiology, Shenzhen Children’s Hospital, Shenzhen, China

**Keywords:** Transcutaneous carbon dioxide partial pressure, End-tidal carbon dioxide partial pressure, Laparoscopic surgery, Pediatric patients, General anesthesia

## Abstract

**Purpose:**

The present study aimed to investigate the correlation between transcutaneous carbon dioxide partial pressure (PtcCO_2_) and arterial carbon dioxide pressure (PaCO_2_) and the accuracy of PtcCO_2_ in predicting PaCO_2_ during laparoscopic surgery in pediatric patients.

**Methods:**

Children aged 2–8 years with American Society of Anesthesiologists (ASA) class I or II who underwent laparoscopic surgery under general anesthesia were selected. After anesthesia induction and tracheal intubation, PtcCO_2_ was monitored, and radial arterial catheterization was performed for continuous pressure measurement. PaCO_2_, PtcCO_2_, and end-tidal carbon dioxide partial pressure (PetCO_2_) were measured before pneumoperitoneum, and 30, 60, and 90 min after pneumoperitoneum, respectively. The correlation and agreement between PtcCO_2_ and PaCO_2_, PetCO_2_, and PaCO_2_ were evaluated.

**Results:**

A total of 32 patients were eventually enrolled in this study, resulting in 128 datasets. The linear regression equations were: PtcCO_2_ = 7.89 + 0.82 × PaCO_2_ (*r*^2^ = 0.70, *P* < 0.01); PetCO_2_ = 9.87 + 0.64 × PaCO_2_ (*r*^2^ = 0.69, *P* < 0.01). The 95% limits of agreement (LOA) of PtcCO_2_ – PaCO_2_ average was 0.66 ± 4.92 mmHg, and the 95% LOA of PetCO_2_ – PaCO_2_ average was –4.4 ± 4.86 mmHg. A difference of ≤ 5 mmHg was noted between PtcCO_2_ and PaCO_2_ in 122/128 samples and between PetCO_2_ and PaCO_2_ in 81/128 samples (*P* < 0.01).

**Conclusion:**

In pediatric laparoscopic surgery, a close correlation was established between PtcCO_2_ and PaCO_2_. Compared to PetCO_2_, PtcCO_2_ can estimate PaCO_2_ accurately and could be used as an auxiliary monitoring indicator to optimize anesthesia management for laparoscopic surgery in children; however, it is not a substitute for PetCO_2_.

**Registration number of Chinese Clinical Trial Registry:**

ChiCTR2100043636.

**Supplementary Information:**

The online version contains supplementary material available at 10.1186/s12887-023-03836-2.

## Introduction

Arterial carbon dioxide pressure (PaCO_2_) is one of the most critical indicators of a patient’s respiratory function. The gold standard for measuring PaCO_2_ in clinical practice is arterial blood gas analysis (ABGA) [1]. The patient’s acid–base balance and electrolyte state can be determined using ABGA. However, arterial blood sampling is an invasive procedure with risks of bleeding, infection, thrombosis, and vascular and neurologic harm. Additionally, ABGA cannot be used to monitor the PaCO_2_ level continuously [2].

End-tidal carbon dioxide partial pressure (PetCO_2_) has become a routine monitoring item for patients undergoing general anesthesia with tracheal intubation. Anesthesiologists can estimate PaCO_2_ based on PetCO_2_. However, several factors, including patient’s age, different types of surgery, combined cardiopulmonary diseases, and changes in pulmonary blood flow, can affect the accuracy of PetCO_2_ monitoring results, increasing the difference between PetCO_2_ and PaCO_2_ in practice [3]. The correlation between PetCO_2_ and PaCO_2_ shows a decrease with the delay of pneumoperitoneum during laparoscopic surgery; thus, PaCO_2_ values should be monitored intermittently by ABGA [4]. Therefore, PetCO_2_ is not a reliable predictor of PaCO_2_.

Transcutaneous carbon dioxide partial pressure (PtcCO_2_) can be used to estimate PaCO_2_. PtcCO_2_ monitoring is based on an electrochemical principle. The probe’s internal heating electrode raises the local skin temperature. This results in the arterialization of dermal capillaries and improvement in their permeability, making it easier for CO_2_ to enter the tissue space and diffuse away from the skin surface. CO_2_ permeates the electrolyte layer via a high-permeability membrane on the sensor’s surface, altering the pH value of the electrolyte layer, which is related to the variation in the CO_2_ partial pressure [5]. The PtcCO_2_ value is obtained by the monitor’s internal programming algorithm. PtcCO_2_ monitoring is a continuous and noninvasive method; however, due to advancements in monitoring technology and the miniaturization of the device, it is gaining increasingly popularity in clinical practice. Also, previous studies have shown that PtcCO_2_ monitoring is effective in perioperative settings [6, 7], which suggests that compared to PetCO_2_, PtcCO_2_ has a better correlation and a smaller difference with PaCO_2_. Nevertheless, Bolliger et al. indicated that PtcCO_2_ monitoring does not accurately reflect PaCO_2_ and does not provide more useful monitoring data than PetCO_2_ [8].

Currently, there are no clinical reports regarding PtcCO_2_ monitoring in pediatric laparoscopic surgery. This study aimed to investigate the correlation and consistency between PtcCO_2_, PetCO_2_, and PaCO_2_ in children who underwent laparoscopic surgery (pneumoperitoneum time > 90 min).

## Methods

The present study was approved by the Shenzhen Children’s Hospital Ethics Committee (Shenzhen, China; Ethics approval number: 202007402), and written informed consent was obtained from the parents. A total of 35 children who underwent laparoscopic surgery, aged 2–8 years, and ASA class Ι or II were recruited for this study. Patients who required a vasoconstrictor to maintain blood pressure during the procedure and those with insufficient pneumoperitoneum time (< 90 min) were excluded from this study.

Children were escorted to the anesthesia room by their parents, and 2.5 mg/kg propofol was administered to induce sleep. The child was then transported to the operating room under the close supervision of the anesthesiologist. Electrocardiogram, pulse oxygen saturation (SpO_2_), and blood pressure (BP)were monitored; the heart rate (HR) and BP were recorded as baseline values. Tracheal intubation was conducted following intravenous administration of benzenesulfonate cisatracurium 0.1 mg/kg and fentanyl 3 μg/kg. Breathing settings were established on the anesthesia machine: intermittent positive pressure ventilation, tidal volume 6–10 mL/kg, inspiration/expiration 1/2, inhalation oxygen concentration 50%, gas flow rate 2 L/min, and respiratory rate 15–25 times/min. PetCO_2_ level was continuously measured using the side-stream capnography (Datex-Ohmeda, Finland, air pumping speed 150 mL/min). The respiratory rate and tidal volume were adjusted to maintain PetCO_2_ 35–45 mmHg and airway pressure 10–25 cmH_2_O. Anesthesia was maintained with inhalation sevoflurane concentration at 2–3%, intravenous pumping of remifentanil 0.2 μg/kg/min, and benzenesulfonate cisatracurium 0.1 mg/kg/h. The dosage of benzenesulfonate cisatracurium was adjusted according to the results of muscle relaxation monitoring.

After tracheal intubation, a PtcCO_2_ monitor (SenTec Digital Monitor, SenTec Inc. Therwil, Switzerland) was attached. The monitoring site was located on the forehead, and the electrode-heating temperature was adjusted to 42 °C. The monitor was calibrated, and the electrode membrane was changed before each use. Radial artery catheterization was performed to facilitate invasive arterial blood pressure monitoring and the acquisition of blood gas analysis samples. The laparoscopic pneumoperitoneum pressure was chosen based on the children's ages (2–4 years old: 9 mmHg, 5–8 years old: 11 mmHg) and it was fine-tuned according to the surgical field's size when the pneumoperitoneum has just been established. Throughout the course of the procedure, there was no change in the pneumoperitoneum pressure. HR and BP were maintained within the range of ± 20% of the baseline value. The nasopharyngeal temperature of the child was maintained at 36–37 °C using an inflatable thermal blanket; the operating room temperature was set at 23–25 °C.

PtcCO_2_ monitor sensor was removed during the postoperative recovery period of anesthesia; the local skin was cleaned and examined for signs of injury. After the removal of the arterial cannula, pressure dressings were applied. The tracheal tube was withdrawn when the child’s spontaneous breathing was recovered, with SpO_2_ maintained at 95% under inhaled air settings. Finally, the vital signs were observed carefully, and the child was transferred to the anesthesia recovery room for further monitoring after stabilization.

ABGA was conducted before (T0) and 30 min (T1), 60 min (T2), and 90 min (T3) after pneumoperitoneum. Also, PtcCO_2_, PetCO_2_, and PaCO_2_ values were recorded at each time point. A blood gas analyzer (i-STAT Analyzer MN: 300-G, Singapore) was used to measure PaCO_2_. HR, BP, SpO_2_, tidal volume, respiratory rate, and body temperature at each time point. HR, BP, and the anesthesia machine’s respiratory parameters were stabilized for at least 5 min before recording the measured values.

Data were analyzed using SPSS version 26.0. Measurement data were presented as mean ± standard deviation (SD). Pearson’s correlation coefficient and linear regression analysis were conducted to establish the correlation, and the Bland–Altman method was utilized to assess the agreement between PetCO_2_ and PaCO_2_ or between PtcCO_2_ and PaCO_2_. A difference of ≤ 5 mmHg between PaCO_2_ and the other two noninvasive variables was clinically acceptable [7, 9] and compared using the chi-square (χ^2^) test. *P* < 0.05 indicated a statistically significant difference.

## Results

Three children were excluded from the research due to the pneumoperitoneum’s short duration (< 90 min), and 32 children (21 men and 11 women; mean age 4.31 ± 1.65 years) were eventually recruited. The average height and weight were 103.63 ± 11.98 cm and 17.45 ± 3.75 kg. Among them, 26 patients underwent laparoscopic pyeloplasty, and 6 patients underwent laparoscopic choledochal cyst excision. A total of 128 datasets were collected, consisting of simultaneous measurements of PtcCO_2_, PetCO_2_, and PaCO_2_ at four time points (Table 1). During the course of the study, no adverse events were observed.

**Table 1:** PtcCO_2_, PetCO_2_, and PaCO_2_ levels at different time points

Variable	T0	T1	T2	T3
PaCO_2_ (mmHg)	35.17 ± 3.06	42.32 ± 3.96	41.51 ± 3.43	40.78 ± 3.44
PtcCO_2_ (mmHg)	35.85 ± 2.98	42.57 ± 3.75	42.26 ± 3.39	41.71 ± 3.43
PetCO_2_ (mmHg)	31.75 ± 2.54	37.28 ± 2.93	36.69 ± 2.61	36.44 ± 2.54

Mean ± standard deviation is used to express the values. T0, T1, T2, and T3 refer to before and 30, 60, and 90 min after pneumoperitoneum, respectively

**Fig. 1:**
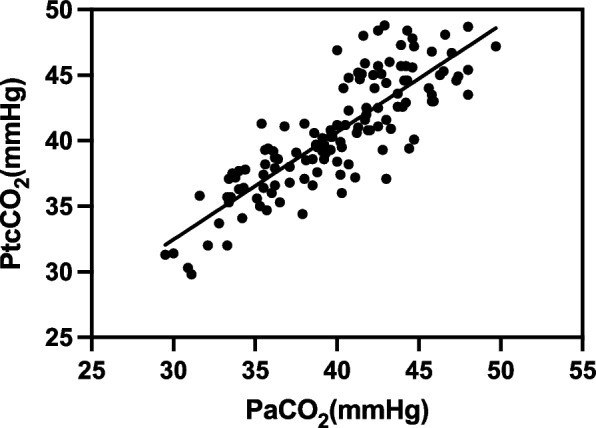
Linear regression analysis between PtcCO_2_ on the y-axis and PaCO_2_ on the x-axis during laparoscopic surgery (pneumoperitoneum time > 90 min) in pediatric patients. The linear regression equation: PtcCO_2_ = 7.89 + 0.82 × PaCO_2_ (*r*^2^ = 0.70, *P* < 0.01, *n* = 128)

The correlation coefficient r between PtcCO_2_ and PaCO_2_ was 0.84 (*P* < 0.01, *n* = 128), and the linear regression equation was PtcCO_2_ = 7.89 + 0.82 × PaCO_2_ (*r*^2^ = 0.70, *P* < 0.01) (Fig. 1). A close correlation was established between PtcCO_2_ and PaCO_2_ at different monitoring time points; however, the correlation decreased slightly with increasing duration of pneumoperitoneum (Table 2).

**Table 2:** Correlation between PtcCO_2_ and PaCO_2_ at different time points

	**T0**	**T1**	**T2**	**T3**
r	0.77	0.76	0.71	0.68
*P*	< 0.01	< 0.01	< 0.01	< 0.01

T0, T1, T2, and T3 refer to before and 30, 60, and 90 min after pneumoperitoneum, respectively

**Fig. 2:**
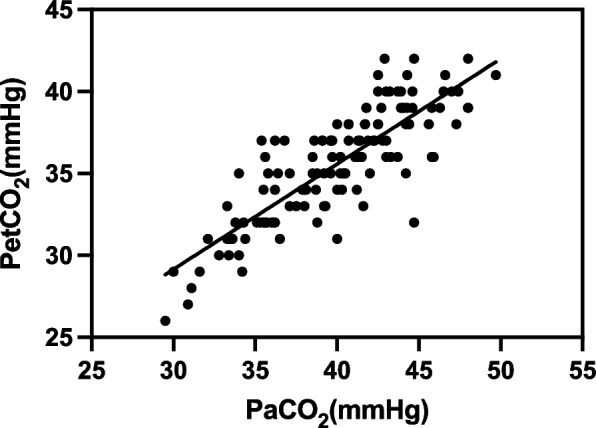
Linear regression analysis between PetCO_2_ on the y-axis and PaCO_2_ on the x-axis during laparoscopic surgery (pneumoperitoneum time > 90 min) in pediatric patients. Linear regression equation: PetCO_2_ = 9.87 + 0.64 × PaCO_2_ (*r*^2^ = 0.69, *P* < 0.01, *n* = 128)

Correlation coefficient r between PetCO_2_ and PaCO_2_ was 0.83 (*P* < 0.01, *n* = 128), and the linear regression equation was PetCO_2_ = 9.87 + 0.64 × PaCO_2_ (*r*^2^ = 0.69, *P* < 0.01) (Fig. 2). A good correlation was established between PetCO_2_ and PaCO_2_ at various time points; however, as the pneumoperitoneum time extended, this correlation decreased significantly compared to PtcCO_2_. The correlation was lowest at T3 (Table 3).

**Table 3:** Correlation between PetCO_2_ and PaCO_2_ at different time points

	**T0**	**T1**	**T2**	**T3**
r	0.87	0.78	0.64	0.58
*P*	< 0.01	< 0.01	< 0.01	< 0.01

T0, T1, T2, and T3 refer to before pneumoperitoneum and 30 min, 60, and 90 min, respectively

**Fig. 3:**
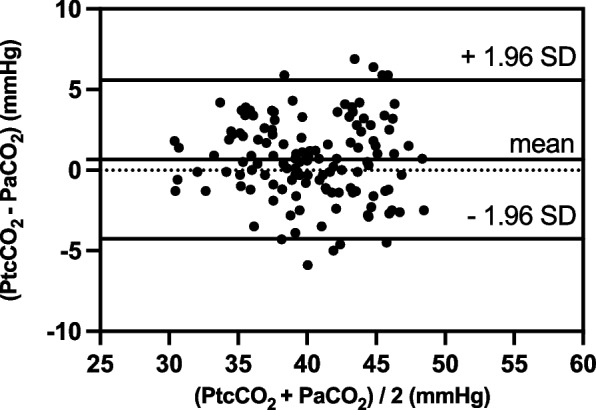
Agreement between PtcCO_2_ and PaCO_2_ was analyzed using the Bland–Altman method. X-axis: **(**PtcCO_2_ + PaCO_2_)/2; Y-axis: PtcCO_2_ – PaCO_2_. The 95% LOA of PtcCO_2_ – PaCO_2_ average was 0.66 ± 4.92 mmHg (mean ± 1.96 SD) (*n* = 128)

**Fig. 4:**
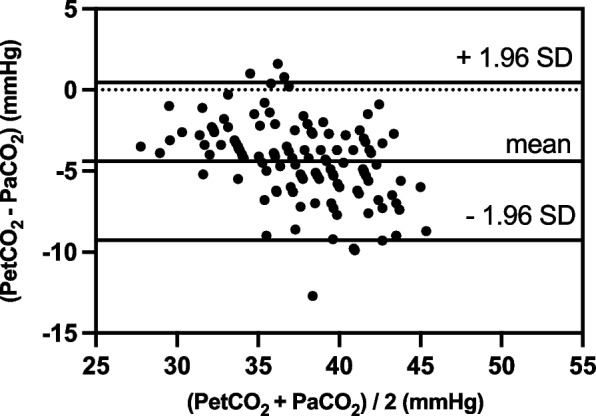
Agreement between PetCO_2_ and PaCO_2_ was analyzed using the Bland–Altman method. X-axis: **(**PetCO_2_ + PaCO_2_)/2; Y-axis: PetCO_2_ – PaCO_2_. The 95% LOA of PetCO_2_ – PaCO_2_ average was –4.4 ± 4.86 mmHg (mean ± 1.96 SD) (*n* = 128)

Difference between PtcCO_2_ and PaCO_2_ was 0.66 ± 2.51 mmHg (*n* = 128). The 95% limits of agreement (LOA) of PtcCO_2_ – PaCO_2_ average was 0.66 ± 4.92 mmHg (mean ± 1.96 SD) (Fig. 3). Difference between PetCO_2_ and PaCO_2_ was –4.4 ± 2.48 mmHg (*n* = 128). The 95% LOA of PetCO_2_ – PaCO_2_ average was –4.4 ± 4.86 mmHg (mean ± 1.96 SD) (Fig. 4). PtcCO_2_ – PaCO_2_ and PetCO_2_ – PaCO_2_ values at each time point are shown in Table 4. A difference of ≤ 5 mmHg was observed between PtcCO_2_ and PaCO_2_ in 122/128 samples and between PetCO_2_ and PaCO_2_ in 81/128 samples (*P* < 0.01).

**Table 4:** PtcCO_2_ – PaCO_2_ and PetCO_2_ – PaCO_2_ values at different time points

Variable	T0	T1	T2	T3
PtcCO_2_ – PaCO_2_ (mmHg) PetCO_2_ – PaCO_2_ (mmHg)	0.69 ± 2.06 –3.42 ± 1.51	0.25 ± 2.66 –5.04 ± 2.50	0.75 ± 2.58 –4.82 ± 2.66	0.94 ± 2.75 –4.34 ± 2.84

Mean ± standard deviation is used to express the values. T0, T1, T2, and T3 refer to before and 30, 60, and 90 min after pneumoperitoneum, respectively

## Discussion

Since the development of minimally invasive surgery, the laparoscopic operation has gained increasingly popular in pediatric surgeries due to its advantages of less trauma, a short hospital stay, less postoperative wound pain, and fewer complications. CO_2_ is the most common gas utilized to create a pneumoperitoneum and provide a good operating view for the surgeon. However, the diffusion capacity of CO_2_ is strong, and the absorption of CO_2_ is sufficient in children due to factors such as the small volume of the abdominal cavity, the proximity of the capillaries to the peritoneum, and the larger abdominal surface area related to weight compared to adults [10]. A risk of hypercapnia is associated with prolonged artificial pneumoperitoneum. Increased CO_2_ alters the body’s acid–base balance and stimulates sympathetic nerves, thus increasing catecholamine and cortisol release and leading to hemodynamic fluctuations [10, 11]. Close monitoring of the CO_2_ level during laparoscopic surgery and timely adjustment of the ventilator parameters is essential to avoid the disruption of physiological functions. PaCO_2_ levels are stabilized after 60 min of pneumoperitoneum [12]; hence, a pneumoperitoneum time of at least 90 min was appropriate to observe the variables in this investigation.

PetCO_2_ is a routine measurement during the perioperative period and one of the primary indicators used to adjust ventilator parameters. However, factors that affect lung ventilation**/**perfusion may interfere with the accuracy of PetCO_2_ measurements, and thus, the use of PetCO_2_ in non-tracheal intubated patients is restricted. The increased abdominal pressure during laparoscopic surgery results in a diaphragmatic rise and an increase in thoracic pressure; subsequently, airway resistance and airway pressure also rise, with pulmonary vasoconstriction and reduced pulmonary blood flow. Pediatric patients are vulnerable to pneumoperitoneal pressure effects. This study revealed that during the entire monitoring process, a good correlation was established between PetCO_2_ and PaCO_2_, *r* = 0.83 (*P* < 0.01). Nevertheless, as the pneumoperitoneum time was prolonged, the correlation between PetCO_2_ and PaCO_2_ decreased gradually, which was consistent with previous findings [6, 9].

Several clinical studies have focused on the application of PtcCO_2_ monitoring in different types of surgery and patients [13–17] under non-tracheal intubation monitoring anesthesia [18, 19]. These studies confirmed the effectiveness of PtcCO_2_ monitoring. The current results showed a close correlation between PtcCO_2_ and PaCO_2_, *r* = 0.84 (*P* < 0.01), and although the correlation was decreased with prolonged pneumoperitoneum time, it was not very significant compared to PetCO_2_. According to Bland–Altman analysis, a lesser mean difference was detected between PetCO_2_ and PaCO_2_ than between PetCO_2_ and PaCO_2_. Therefore, PtcCO_2_ performed better than PetCO_2_ in estimating PaCO_2_, which is in agreement with the previous results [6, 7, 9]. In our experiment, we can combine PetCO_2_, PaCO_2_, and PtcCO_2_ to regulate the patient's acid–base, so there is no accumulation of CO_2_ during the whole operation. However, due to the limitations of PetCO_2_ monitoring, especially in the case of prolonged pneumoperitoneum, relying solely on PetCO_2_ to regulate the patient's respiratory parameters cannot guarantee that the patient is in acid–base balance, particularly for young children. And PtcCO_2_ can more accurately estimate PaCO_2_, so its application can reduce the risk of CO_2_ accumulation. Conway et al. conducted a meta-analysis on the effectiveness of PtcCO_2_ monitoring [20] and demonstrated that is challenging to achieve a uniform standard due to the involvement of various clinical aspects, including the monitoring site, electrode heating temperature, and application population; thus, it is critical to monitor the PtcCO_2_ trend throughout the monitoring process.

The CO_2_ level measured by PtcCO_2_ monitoring consists of two parts: one derived from the blood (arterial, capillary, and venous), and the other from the metabolism of the tissue cells [21, 22]. The warming effect of the electrode increases the skin blood flow and enhances the contribution of arterial blood to CO_2_ by opening the precapillary sphincter [23]. A rise in the local skin temperature increases the metabolism of tissue cells, producing excessive CO_2_. Therefore, the PtcCO_2_ monitoring value is theoretically higher than that of PaCO_2_. PtcCO_2_ monitors used in clinical practice correct the initial measured value based on the selected heating temperature to reduce the deviation from PaCO_2_ [21]. In the present study, PtcCO_2_ monitoring values were less than PaCO_2_ in 51/128 data sets; hence, the correction method for PtcCO_2_ monitors needs to be investigated further.

Several factors affect PtcCO_2_ monitoring, including the temperature of the electrodes, the monitoring location of the sensor, and the patient’s clinical state. Nishiyama et al. demonstrated that when the anterior chest (between the clavicle and nipple) was chosen as the monitoring site, PtcCO_2_ correlated best with PaCO_2_ at 43 °C (*R*^2^ = 0.7568) among the different electrode-heating temperatures (37, 40, 42, 43, and 44 °C) in its setting, and the monitoring required less time to stabilize at higher temperatures as blood CO_2_ levels change, but required > 150 s [24]. According to a study on the optimal electrode temperature for monitoring PtcCO_2_ in preterm infants, the mean difference between PtcCO_2_ and PaCO_2_ was the smallest at 42 °C [25]. A higher temperature may result in skin damage in pediatric patients due to thin skin, but previous studies have not reported any skin injuries in children or infants. In this study, we chose 42 °C as the electrode temperature for PtcCO_2_ monitoring; no adverse events were observed.

Nishiyama et al. reported that PtcCO_2_ was correlated with PaCO_2_ when the monitoring sensor was located on the chest (*R*^2^ = 0.76) but not when it was located on the upper arm and forearm (R^2^ < 0.5) [26]. When the anterior chest is chosen as a monitoring site in pediatric patients, the area of surgical disinfection might be affected, especially in younger kids. Anesthesiologists were usually positioned on the cephalic side of the patient, such that the forehead was selected as the site in this study, facilitating the administration of the probe. In the current study, PetCO_2_ showed a close correlation with PaCO_2_ than PtcCO_2_ before pneumoperitoneum; however, the mean difference between PtcCO_2_ and PaCO_2_ was smaller than the mean difference between PetCO_2_ and PaCO_2_. However, whether PetCO_2_ correlates better with PaCO_2_ than PtcCO_2_ in pediatric patients under non-pneumoperitoneal conditions with the forehead as the monitoring site needs to be studied further with a large sample size.

In the event that patients’ peripheral tissues and organs are not supplied adequately with blood, such as in shock, the CO_2_ produced by tissue metabolism cannot be carried away quickly, and PtcCO_2_ monitoring values increase gradually [22]. Thus, PtcCO_2_ can be utilized as one of the indicators for assessing a patient’s microcirculatory status, which is useful in guiding the treatment [27]. However, the study on PtcCO_2_ monitoring in surgical patients with circulatory failure has been rarely reported, and the correlation between the PtcCO_2_ gradient changes and skin tissue perfusion status requires further clinical investigation. Other factors, such as poor skin contact with the fixed connection loop and insufficient gel, may allow contact between the probe and air, thus interfering with the monitoring results. CO_2_ permeability films that have not been replaced for a long time or are damaged or air bubbles under the film can also affect the accuracy of PtcCO_2_ monitoring.

Since PtcCO_2_ monitoring is a continuous and noninvasive method that can be used to assess PaCO_2_ to some extent, its perioperative application is promoted in the different types of surgery and populations. Endotracheal intubation is not required for gastrointestinal endoscopy or other operations that can be performed using nerve blocks. The use of intravenous anesthetic medications intraoperatively can improve operating conditions and increase patient comfort during these procedures. When the nerve block is unsatisfactory, or when specific operations call for an enhanced level of sedation, supplemental narcotic medicines are required. Understanding the CO_2_ level of patients allows us to more precisely regulate the intravenous anesthetic medicine dosage. However, it is often difficult to accurately monitor the CO_2_ levels of patients during these operations. In this situation, PtcCO_2_ monitoring is a good choice. It has been shown [18, 19, 28] that PtcCO_2_ monitoring is an effective way to detect hypoventilation in patients, which reduces the incidence, extent, and duration of hypercapnia, improving the safety of patients under sedation. High-frequency ventilation is often used to maintain oxygenation in some airway procedures performed with a rigid bronchoscope. However, evaluating the ventilatory status of patients with PetCO_2_ in the open ventilation mode of high-frequency ventilation is challenging. As a result, we can adjust the parameters of high-frequency ventilation to avoid the accumulation of CO_2_ according to PtcCO_2 _[29].

Usually, patients undergoing thoracic surgery have chronic lung diseases and require one-lung breathing in the lateral decubitus position during operation. These factors can affect lung ventilation/perfusion, leading to the increase of the difference between PetCO_2_ and PaCO_2_, and patients are more likely to develop respiratory acidosis. Oshibuchi M's study [30] showed that PtcCO_2_ can more accurately predict PaCO_2_ compared with PetCO_2_ in both two-lung ventilation and one-lung ventilation. It has been shown [31] that PtcCO_2_ monitoring remains highly accurate even when one-lung ventilation is prolonged (more than 2 h) and permissive hypercapnia is present.

Most patients need to recover in anesthesia recovery room after surgery. Due to the presence of residual opioids and muscle relaxants, patients are at potential risk of developing respiratory depression, especially in elderly and obese patients. PtcCO_2_ monitoring effectively reflects PaCO_2_ levels and is more suitable for observing changes in CO_2_ fluctuations over time so that we can take appropriate treatment measures [32]. For pediatric patients, the anesthesia has certain particularity. Children are often unable to cooperate with us for some examination operations. They must be under sedation and analgesia conditions prior to nerve block or spinal anesthesia. Children must maintain a certain level of sedation throughout the whole operation, but the use of anesthetic drugs will always have an impact on their breathing more or less. By using PtcCO_2_ monitoring, anesthesiologists are able to determine in time whether CO_2_ accumulation in patients is occurring so that the appropriate treatment can be administered.

PtcCO_2_ monitoring also has some limitations. The monitoring site should be cleaned in advance to remove the hair and grease; also, the PtcCO_2_ monitor requires a calibration time of approximately 15 min before use and needs to be recalibrated either after the patient is removed from the monitoring site or after prolonged monitoring. When CO_2_ in the blood changes, PtcCO_2_ monitoring takes about 2 min to reflect PaCO_2_ with a degree of delay [33]. These factors limit its use in surgery patients. Therefore, the PtcCO_2_ monitor needs further improvement to facilitate its use during the perioperative period. Some studies have reported that PtcCO_2_ monitoring techniques are not based on electrochemical principles [34–36]. Because of the different monitoring mechanisms, the local heating on the skin is avoided, and the time required for calibration and stabilization is short, rendering the monitors convenient to use. However, the application is still not mature in clinical practice. Although PetCO_2_ is susceptible to various factors, it plays an essential role in determining the position of the tracheal tube, tube folding, and accidental decannulation [37]. Additionally, PetCO_2_ provides information about the patient’s pulmonary blood flow status and circulatory function [38], and the patient’s airway status can also be determined from the PetCO_2_ waveform, thereby deeming that PtcCO_2_ is not a substitute for PetCO_2_.

In conclusion, PtcCO_2_ shows a close correlation with PaCO_2_ when the forehead is chosen as a monitoring site in children undergoing laparoscopic surgery. Compared to PetCO_2_, PtcCO_2_ can accurately estimate PaCO_2_ and could be used as an auxiliary monitoring indicator to optimize anesthesia management for laparoscopic surgery in children; however, it is not a substitute for PetCO_2_.

## Supplementary Information

**Additional file 1**.

## Data Availability

All data and material generated or analysed during this study are included in this published article and its supplementary information files. And the datasets used and/or analysed during the current study are also available from the corresponding author upon reasonable request.
